# Anterior Ischemic Optic Neuropathy in a Child Receiving Chronic Hemodialysis

**DOI:** 10.1155/2020/7012586

**Published:** 2020-03-16

**Authors:** Joyce Moore, Jennifer Klowak, Gloria Isaza, Steven Arora, Vladimir Belostotsky, Nina Stein, Rahul Chanchlani

**Affiliations:** ^1^Department of Medicine, Michael G. DeGroote School of Medicine, McMaster University, Hamilton, Ontario, Canada; ^2^Department of Pediatrics, McMaster Children's Hospital, Hamilton, Ontario, Canada; ^3^Department of Ophthalmology, McMaster University, Hamilton, Ontario, Canada; ^4^Division of Nephrology, Department of Pediatrics, McMaster Children's Hospital, Hamilton, Ontario, Canada; ^5^Department of Radiology, McMaster University, Hamilton, Ontario, Canada

## Abstract

Anterior ischemic optic neuropathy (AION) occurs due to hypoperfusion of the optic nerve and is a rare complication in patients receiving maintenance dialysis. To date, AION has only been reported in 22 children, all of whom were receiving peritoneal dialysis. We report the first case of AION in a 2-year 11-month-old child receiving chronic hemodialysis secondary to polycystic kidney disease from a phosphomannomutase 2 gene mutation. This case highlights the consideration for frequent blood pressure monitoring and ophthalmic screening in a certain cohort of children receiving chronic dialysis.

## 1. Background

Anterior ischemic optic neuropathy (AION) is characterized by hypoperfusion of the posterior ciliary arteries resulting in optic nerve infarction [1]. AION has been reported as a complication of hypotension secondary to chronic dialysis in adult patients, but it is much less common in children [2]. To our knowledge, only 22 cases of pediatric AION have been reported and all of them were receiving peritoneal dialysis [1, 3–5]. We present what we believe to be the first case report of AION in a child receiving chronic intermittent hemodialysis.

## 2. Case Report

We report a 2-year 11-month-old male on chronic hemodialysis secondary to polycystic kidney disease (PCKD) who underwent bilateral nephrectomy. He had a compound heterozygous phosphomannomutase 2 promoter and missense mutation, accounting for PCKD and congenital hyperinsulinism. His left kidney was removed in February 2017 due to respiratory insufficiency from mass effect. Seven months later, with significantly reduced kidney function, he underwent a left nephrectomy due to respiratory compromise and hypertension. Following his second nephrectomy, he began intermittent hemodialysis five days a week; decreased to four days a week after 6 months.

Prior to his second nephrectomy, he was being treated for hypertension with an angiotensin-converting enzyme inhibitor, beta-blocker, and furosemide. After the second nephrectomy, his intradialytic systolic blood pressure used to be around 68 mmHg. For this reason, he was started on 2.5 mg midodrine during hemodialysis for systolic blood pressure below 90 mmHg. His ultrafiltration was minimized to avoid further hypotensive episodes during hemodialysis. Despite that, his systolic blood pressure stayed around 60 mmHg. Other medications included diazoxide, omeprazole, levothyroxine, and darbepoetin. He was G-tube fed without salt restriction. His blood work five days prior to presentation demonstrated normocytic, mild anemia (hemoglobin 95 g/L) with a reticulocyte count of 20 × 10^9^/L. His electrolytes were grossly normal (sodium 136 mmol/L, potassium 3.8 mmol/L, chloride 91 mmol/L, and calcium 2.77 mmol/L) and his albumin was low at 30 g/L. Creatinine was elevated at 421 *μ*mol/L. His echocardiogram showed normal biventricular function.

In June 2018, 9 months after initiating hemodialysis, he presented to the emergency department with an unsteady gait and difficulty focusing and following objects starting approximately two hours after completion of hemodialysis that day. Parents noted a pupil size change from small to large bilaterally, documented at 8 mm. On his initial neurological examination, his pupils were dilated but responsive, and he had poor visual acuity and an unsteady gait. He was otherwise alert, oriented, and comfortable. Vitally, his blood pressure was 59/33 mmHg, heart rate was 92 bpm, and he was afebrile. Laboratory findings on admission demonstrated a normocytic, mild anemia with a hemoglobin level of 101 g/L and an MCV of 92.7 fL. His sodium was found to be low at 127 mmol/L, with a potassium level of 4.4 mmol/L, and a chloride level of 98 mmol/L. Repeat blood work displayed a sodium level of 134 mmol/L, and thus, no hypertonic saline was administered. Creatinine was elevated at 166 *μ*mol/L. Tox screen was negative, and AST, ALT, and INR were all normal. He had no history of trauma, toxin ingestion, sick contact exposure, or seizure activity.

**Figure 1:**
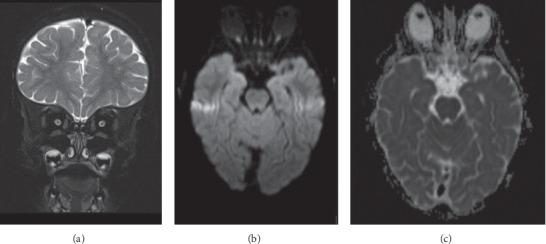
Brain MRI at patient's presentation. Coronal T2 turbo spin echo with fat saturation of the orbits showing normal thickness and T2 signal of the optic nerves. No asymmetry noted (a). Diffusion weighted imaging B1000 (b) and apparent diffusion coefficient map (c). The visualization of the optic nerves is distorted by the presence of artifacts, and allowing for this limitation, no restricted diffusion was observed on the apparent diffusion coefficient map.

Head CT displayed nonspecific findings of bifrontal subcortical white matter hypodensities. MRI displayed multiple foci of increased fluid-attenuated inversion recovery signal, mainly on bilateral subcortical white matter of the frontal lobes and corona radiata. Optic nerve size and signal intensity were normal (Figure 1). Ophthalmology noted decreased visual acuity to hand motion and optic disk pallor, and diagnosed optic nerve atrophy. He was treated with a five-day course of intravenous pulse methylprednisolone (15 mg/kg, 225 mg daily). Enteral midodrine was administered three times a day, and fluids were titrated for a blood pressure goal above 70/40 mmHg.

**Figure 2:**
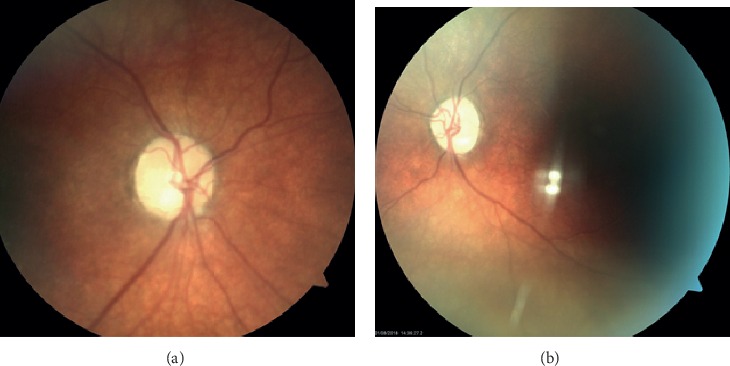
Fundoscopic images of retina from two-month follow-up. The left optic nerve (a) is more pale than the right (b). Vision at this time was 20/800 in the left eye and 20/30 in the right eye.

He was discharged after steroid treatment with a diagnosis of bilateral AION. Ophthalmology follow-up two months after his acute presentation revealed his vision to be 20/800 in the left eye and 20/30 in the right eye (Figure 2). He continued midodrine until his related living donor kidney transplant. He is currently stable and normotensive six months posttransplant. His vision at 10 months following his initial presentation is 20/40 in the right eye and no light perception in the left.

## 3. Discussion

AION results from poor perfusion of the optic nerve and is a rare but serious condition that can occur in patients on chronic dialysis [2]. The optic nerve is supplied by the posterior ciliary arteries which arise from the ophthalmic artery [3]. Compromised blood flow through these arteries may lead to ischemia of the optic nerve [3, 5]. If adequate blood flow is not promptly restored, permanent vision loss can occur [3, 5]. Many factors can contribute to hypoperfusion of the optic nerve, and the risk factors vary between adults and children [3, 5]. In adults, AION is classified as either arteritic or nonarteritic [6]. The former is commonly associated with giant cell arteritis and results due to thromboembolism in the posterior ciliary arteries [6]. The latter is more common in adults and occurs due to risk factors such as diabetes, vasospasm, nocturnal hypotension, and sleep apnea [6]. On the other hand, children do not have risk factors associated with aging, and infarction of the optic nerve is more commonly related to systemic hypotension [3]. Risk factors in children include young age at dialysis onset, chronic hypotension (often with previous hypertension), history of bilateral nephrectomy, autosomal recessive polycystic disease, and anemia [1, 2, 5].

To date, 22 cases of AION have been reported in children, all of whom were on peritoneal dialysis. Similarities of our case with the previously reported 22 cases are the bilateral nature of vision loss in 95% (21/22) of patients and sudden presentation in all patients [1, 3–5]. As with our case, 77% (17/22) of the cases also presented with hypotension, further supporting systemic hypotension as a risk factor for AION. The mean age of patients was 3.3 years. Of the 21 patients available for follow-up, 62% (13/21) had partial vision recovery, as did our patient, and 38% (8/21) remained blind [1, 3–5]. The optic disc pallor seen at presentation in our case has been reported previously in acute pediatric AION related to dialysis [3]. Unique features are that this is the first reported case of pediatric AION during hemodialysis and that it was bilateral, whereas adult AION following hemodialysis typically presents with monocular loss. However, AION following peritoneal dialysis in both children and adults is usually a bilateral disease [3]. This suggests that the mechanism for AION in children may be similar in both hemo- and peritoneal dialysis patients and highlights the need for better screening across all patients.

This case emphasizes the importance of blood pressure control in children receiving chronic hemo- or peritoneal dialysis. Dialysis patients have frequent contact with the health care system, providing ample opportunity for blood pressure monitoring and management. All children with risk factors for AION, such as anemia, young age, chronic hypotension, bilateral nephrectomy, and polycystic kidney disease, should be regularly screened for AION with an early involvement of ophthalmology as needed. Adequate medical management of low blood pressure and early diagnosis of AION could reduce both the incidence of AION and the associated potential for complete vision loss in patients receiving chronic dialysis.
